# Supplementary material for: Bioengineered Silkworm for Producing Cocoons with High Fibroin Content for Regenerated Fibroin Biomaterial-Based Applications
Source: Int J Mol Sci. 2022 Jul 4;23(13):7433. doi: 10.3390/ijms23137433 (PMC9267247; doi:10.3390/ijms23137433)
Supplement: Supplementary file 1 [file ijms-23-07433-s001.zip › ijms-1753627-supplementary.pdf]

Supplemental Materials

Bioengineered Silkworm for Producing Cocoons with High Fibroin Content for Regenerated Fibroin Biomaterial-Based Applications

Mana Yamano [1,†], Ryoko Hirose [1,†], Ping Ying Lye [1], Keiko Takaki [1,2], Rina Maruta [1], Mervyn Wing On Liew [3], Shinichi Sakurai [4,5], Hajime Mori [1] and Eiji Kotani [1,2,*]

[1] Department of Applied Biology, Kyoto Institute of Technology, Sakyo-ku, Kyoto 606-8585, Japan; manakichy.y@gmail.com (M.Y.); hrro0621@gmail.com (R.H.); ying5434@hotmail.com (P.Y.L.); ktakaki@kit.ac.jp (K.T.); rina.maruta07@gmail.com (R.M.); silk7776@gmail.com (H.M.)
[2] Biomedical Research Center, Kyoto Institute of Technology, Sakyo-ku, Kyoto 606-8585, Japan
[3] Institute for Research in Molecular Medicine, Universiti Sains Malaysia, Minden, Penang 11800, Malaysia; mervynliew@yahoo.com
[4] Department of Biobased Materials Science, Kyoto Institute of Technology, Sakyo-ku, Kyoto 606-8585, Japan; shin1sakuri@gmail.com
[5] Department of Chemical Engineering, Indian Institute of Technology Guwahati, Guwahati 781039, Assam, India
* Correspondence: kotani@kit.ac.jp; Tel.: +81-75-724-7774
† These authors contributed equally to this work.

1. Supplementary Materials and Methods

1.1. Vector construction

All plasmids constructed in this study were confirmed by DNA sequencing. The oligonucleotide primer sequences are listed in Supplementary Table S1. For vector construction, PCR was performed using Q5 High-Fidelity DNA polymerase (New England Biolabs, Ipswich, MA, USA). We used a plasmid containing a nucleotide sequence encoding the protein designated P1A269 in previously reported pIZ-H1/P1A269/FLAG with an N-terminal cypovirus polyhedrin H1 and C-terminal FLAG (DYKDDDK) for immunological detection using antibodies [20].

1.1.1. Plasmid for Ser1-P1A269 line

The promoter and 5′-untranslated region of silkworm *Ser1* (at positions -579 to +59; accession no. DQ354392) [29] was amplified using PCR from *w1-pnd* genomic DNA with primers ser1fKpnI and ser1rBamHI, digested with restriction enzymes, and then inserted into the KpnI-BamHI restriction site of pIZ-H1/P1A269/FLAG by ligation using Ligation High version 2 (TOYOBO, Osaka, Japan). To construct the donor plasmid pBacMCS[Ser1Pro-H1/P1A269/FLAG, 3xP3-EGFP] for silkworm transgenesis (Figure 1A), the fusion sequence for P1A269 along with the upstream Ser1 promoter and downstream OPIE2 3′ noncoding region derived from pIZ/V5-His (Thermo Fisher Scientific, Waltham, MA, USA) was

amplified by PCR using primers In-fusion-fSer1 and In-fusion-r1. The amplified fragment was inserted into the BlnI site of pBacMCS[UAS, 3xP3-egfp] [10, 11, 20, 38] using an In-fusion HD Cloning Kit (Takara Bio, Shiga, Japan) in accordance with the manufacturer's instructions. Plasmid DNA with the correct insertion was purified using a Plasmid Midiprep Kit (QIAGEN, Hilden, Germany).

1.1.2. Plasmid for Ser3-P1A269 line

The promoter and 5′-untranslated region of silkworm *Ser 3* (at the nucleotide position of –571 to +21) [30] were amplified by PCR from *w1-pnd* genomic DNA with primers ser3fKpnI and ser3rBamHI, digested with a restriction enzyme, and then inserted into the KpnI–BamHI restriction site of pIZ-H1/P1A269/FLAG by ligation using Ligation High version 2 (TOYOBO). To construct the donor plasmid pBacMCS[Ser3Pro-H1/P1A269/FLAG, 3xP3-DsRed] for silkworm transgenesis (Figure 1B), the fusion sequence for P1A269 along with the upstream *Ser3* promoter and downstream OPIE2 3′ noncoding region derived from pIZ/V5-His (Thermo Fisher Scientific) was amplified by PCR using primers In-fusion-fSer3 and In-fusion-r2, and the synthesized fusion sequence for DsRed along with the upstream 3xP3 promoter and downstream SV40 [30] was amplified by PCR using the primers In-fusion-f3xP3 and In-fusion-rSV40. The two DNA fragments were sub-cloned into linearized pBacMCS[UAS, 3xP3-egfp] [10,11,20,38] and digested with EcoRI and BglII using the In-fusion HD Cloning Kit (Takara Bio) in accordance with the manufacturer's instructions. Plasmid DNA with the correct insertion was purified using a Plasmid Midi Prep Kit (QIAGEN).

1.2. Generation of the transgenic silkworms

To generate transgenic silkworms, DNA was microinjected into silkworm eggs as previously described [40,41]. To generate the Ser1-P1A269 line, donor plasmid, pBacMCS[Ser1Pro-H1/P1A269/FLAG, 3xP3-EGFP], and helper plasmid expressing transposase were injected into the *w1-pnd* embryo at the pre-blastoderm stage. In the next generation, individuals with eyes and nervous systems expressing the vector-derived marker trait EGFP were screened. Marker-positive individuals (individuals with EGFP-positive eyes and nervous system) in the succeeding generations were sib-mated, and a transgenic line carrying homogeneous chromosomes with the transgene Ser1-P1A269 was established.

In addition, to generate the Ser3-P1A269 line, pBacMCS [Ser3Pro-H1/P1A269/FLAG, 3xP3-DsRed], and the helper plasmid were microinjected into silkworm eggs in a similar manner. Marker-positive individuals (individuals with DsRed-positive eyes and nervous systems) in succeeding generations were sib-mated, and the transgenic line, Ser3-P1A269, was established. Homogeneous Ser1-P1A269 and Ser3-P1A269 lines were kept through more than 15 generations of sib mating.

1.3. Inverse-PCR methods for analyzing border Bombyx mori *sequences at the transgene positions*

Genomic DNA from the silk glands of the fifth instar larvae of each marker-positive silkworm line was purified using the standard phenol-chloroform extraction method,

incubated overnight with proteinase K, digested with Sau3AI, and circularized by overnight ligation at 16 °C. DNA fragments were amplified from the circularized template by PCR amplification from both the upstream and downstream inverted terminal repeats of the *piggyBac* sequence using primers (Supplementary Table S1) and sequenced.

1.4. Detection of P1A protein expressed in the middle silk gland by western blotting

Middle silk glands (MSGs) were collected from Ser1-P1A269 or Ser3-P1A269 fifth instar larvae, along with *w1-pnd*, and homogenized in RIPA buffer containing a proteinase inhibitor (Nacalai Tesque, Kyoto, Japan). Proteins (5 µg in each lane) in the homogenates were further heat-treated in 1x SDS-PAGE sample buffer, separated on a 12.5% gel, and transferred onto a polyvinylidene fluoride membrane. The membrane was incubated with 1:5,000 anti-FLAG® M2 peroxidase (horseradish peroxidase) mouse monoclonal antibody (Sigma-Aldrich, St. Louis, MO, USA) diluted in PBS(-) containing 5% Blocking One (Nacalai Tesque). The horseradish peroxidase signal was developed using Chemi-Lumi One L (Nacalai Tesque) in accordance with the manufacturer's instructions.

1.5. Analysis of amino acid composition of cocoons and commercial silk threads

The amino acid composition of hydrolyzed cocoon samples (3.2 mg proteins) from cocoons of the Ser1-P1A269 line, *w1-pnd* silkworm, or *w1-pnd*$^{P1A269/P1A269}$ [20] line, along with commercial silk fiber prepared by degumming, with the specific artificial condition for preparing threads solely composed of fibroin, was analyzed using Prominence high-performance liquid chromatography (Shimadzu Corporation, Kyoto, Japan).

2. Supplementary Figures

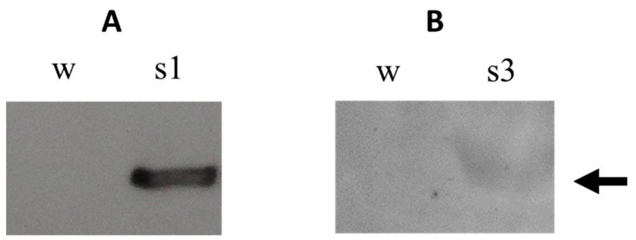

Figure S1. Recombinant P1A269 protein expression in MSGs of day 5 Ser1-P1A269 (s1; **A**) and day 6 Ser3-P1A269 (s3; **B**) fifth instar larvae. P1A269 protein with C-terminal FLAG tag was detected in the MSG protein samples by western blotting using FLAG antibody as described in the Supplementary Materials and Methods. Protein samples of MSGs of non-transgenic *w1-pnd* line (w) on the corresponding day of fifth instar were applied as negative controls.

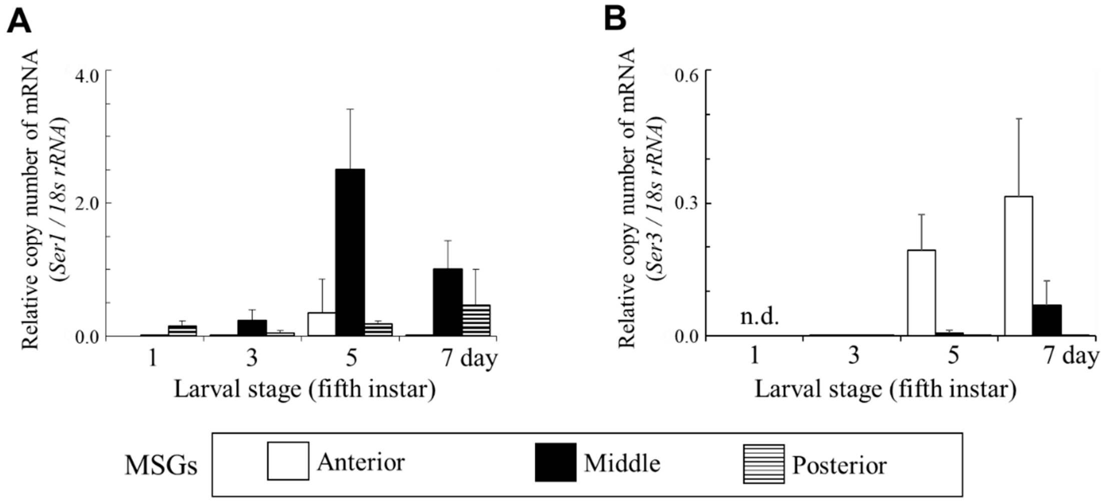

Figure S2. Quantitative RT-PCR analysis of Ser1 (**A**) or Ser3 (**B**) in anterior, middle, and posterior regions of MSGs of *w1-pnd* on the indicated day of fifth instar. The mRNA levels of Ser1 and Ser3 were quantified and normalized to that of 18S ribosomal RNA. Data are shown as the means ± SD of relative values obtained (copy number); *n* = five; n.d., not detected.

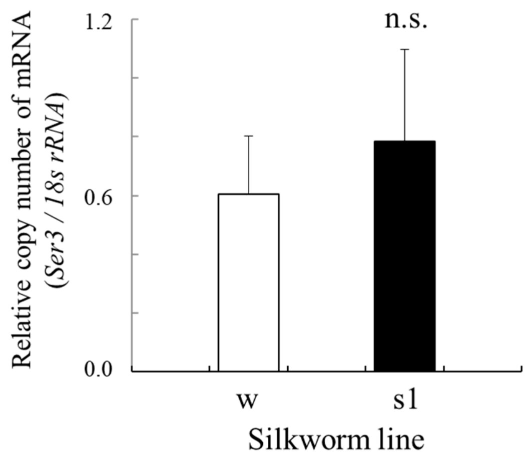

Figure S3. Quantitative RT-PCR analysis of Ser3 in the MSGs of Ser1-P1A269 larvae. The mRNA levels of Ser3 were quantified and normalized to that of 18S ribosomal RNA. Data are shown as the means ± SD of relative values obtained (copy number); *n* = five; n.s., not significant (Student's *t*-test).

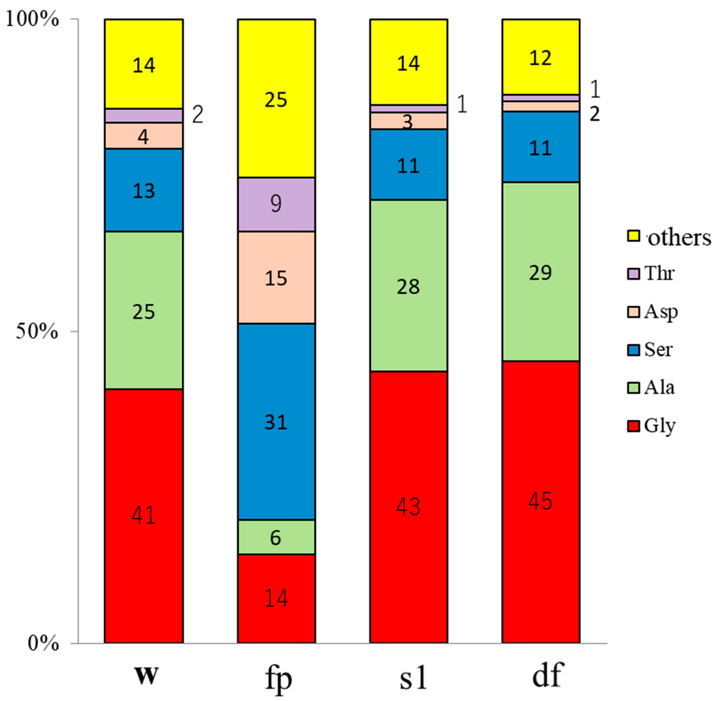

Figure S4. Comparison of amino acid composition between cocoons from *w1-pnd* (w), Ser1-P1A269 line (s1), and commercial silk thread fibroin (df) prepared by degumming, with the specific artificial condition for preparing threads solely composed of fibroin. Transgenic sericin cocoons from *w1-pnd*$^{P1A269/P1A269}$ line [20], which express P1A269 in the PSGs under the control of FibH promoter (fp).

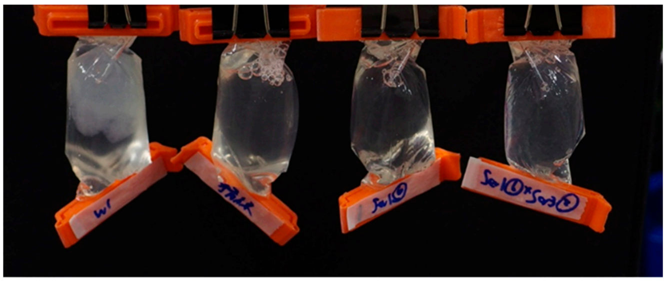

w dw s1 s13

Figure S5. Precipitates emerged in the dialysis bags during the dialysis step for fibroin isolation from non-degummed cocoons of *w1-pnd* (w), Ser1-P1A269 (s1), and Ser1/3-P1A269 (s13), along with degummed cocoons of *w1-pnd* (dw).

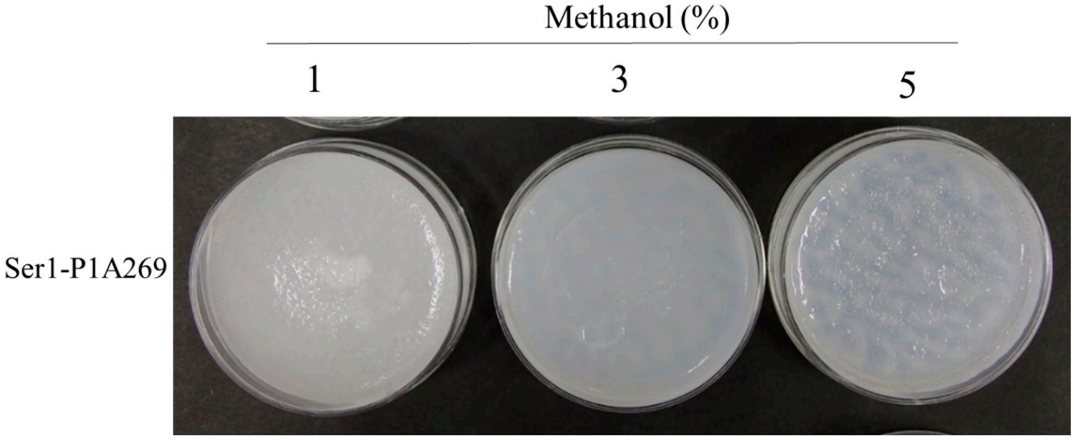

Figure S6. Sponges prepared by adding increasing volume percentages of methanol (1%, 3%, and 5%) to isolated fibroin solutions from Ser1-P1A269 cocoons.

A

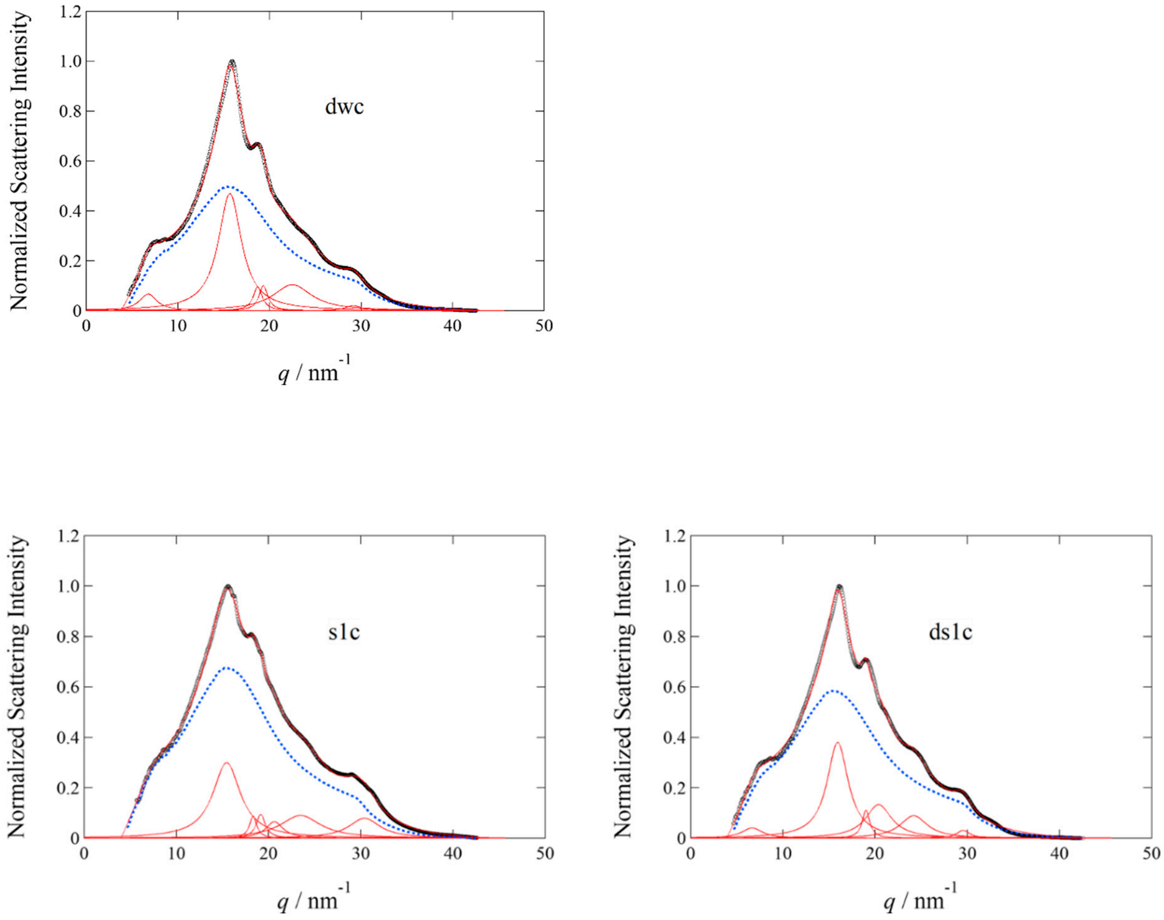
dwc
s1c
ds1c
Normalized Scattering Intensity
q / nm-1
0
0.2
0.4
0.6
0.8
1.0
1.2
10
20
30
40
50

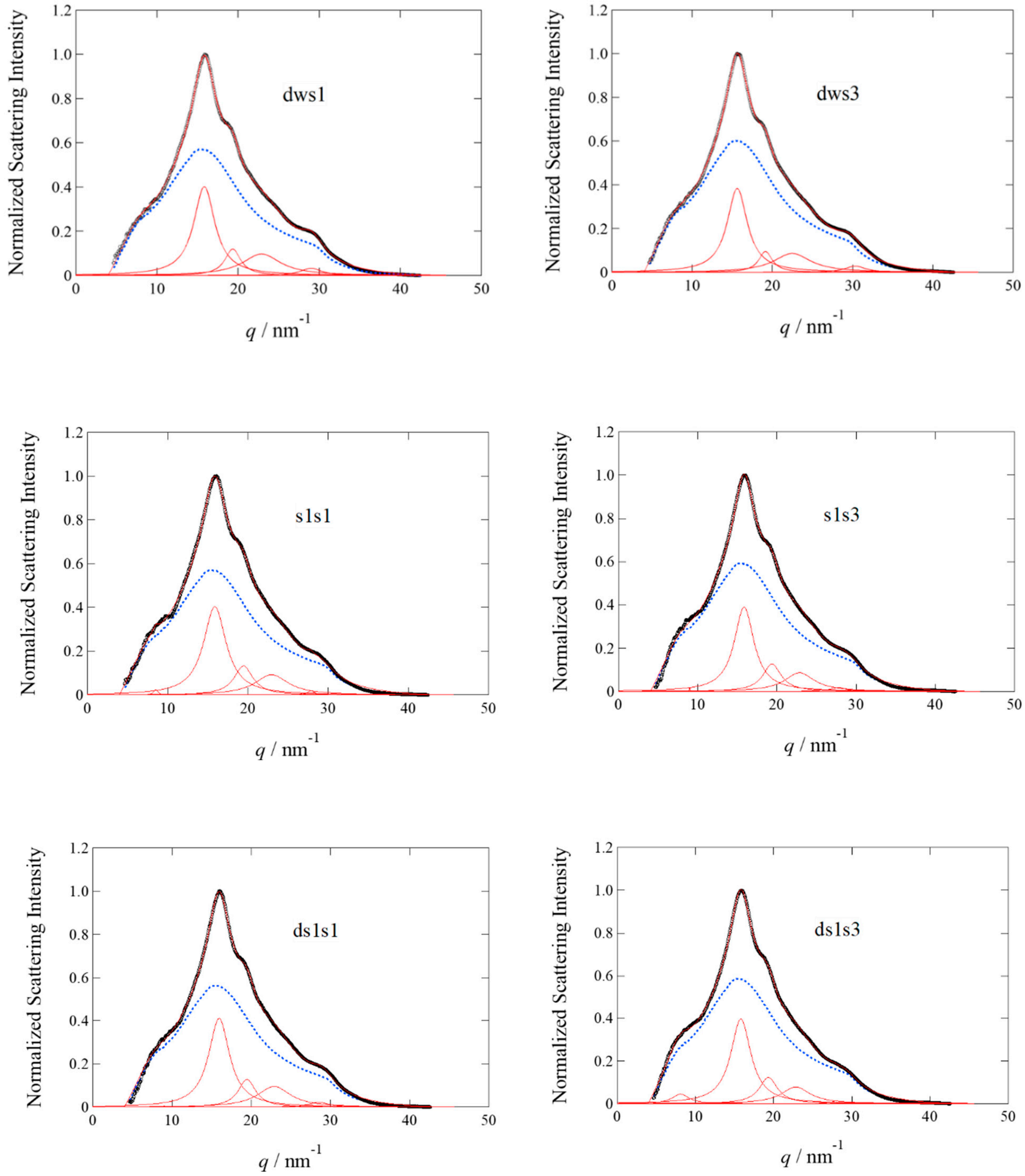
dws1
dws3
s1s1
s1s3
ds1s1
ds1s3
Normalized Scattering Intensity
q / nm-1
0
0.2
0.4
0.6
0.8
1.0
1.2
10
20
30
40
50

B

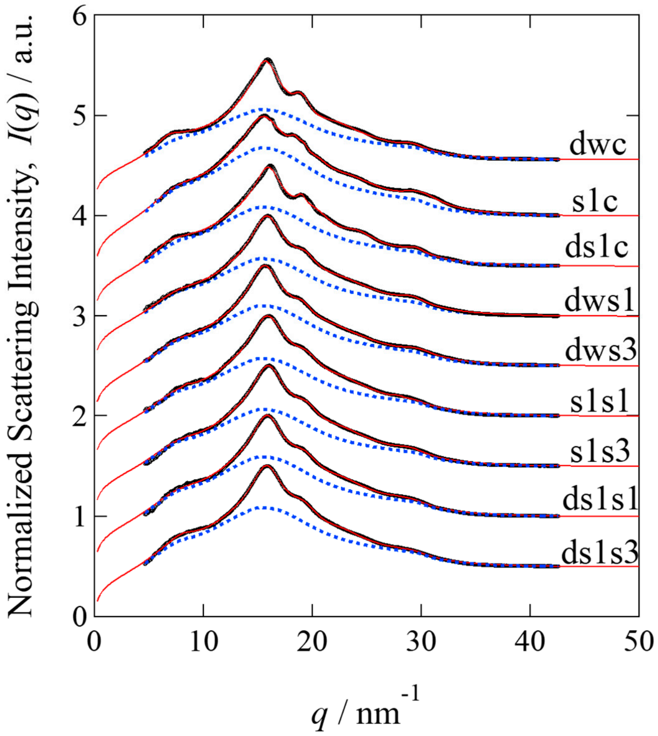

Figure S7. (**A**) Results of peak deconvolution of the measured normalized WAXD profiles of all samples. The black open circles indicate the measured normalized WAXD profiles, red curves are deconvoluted peaks (mathematically they are assumed as the Lorentz function), and blue broken curve is the contribution of the amorphous halo, which was appropriately determined by multiplication to the WAXD profile of the amorphous sample with the fraction determined by the least-square parameter fitting using the Lorentz peaks. The summation (re-construction) of all deconvoluted peaks (red curves) with the amorphous contribution (blue broken curve) is shown with the red curve on the measured normalized WAXS profile, indicating an almost perfect fit to the measured WAXD profile. The samples examined are lyophilized degummed *w1-pnd* cocoon (dwc), Ser1-P1A269 cocoon (s1c), degummed Ser1-P1A269 cocoon (ds1c), sponge from degummed *w1-pnd* fibroin added 1% (dws1) or 3% (dws3) methanol, sponge from Ser1-P1A269 fibroin added 1% (s1s1) or 3% (s1s3) methanol, and sponge from degummed Ser1-P1A269 fibroin added 1% (ds1s1) or 3% (ds1s3) methanol. (**B**) Summary of normalized one-dimensional WAXD profiles for all samples with fitted amorphous profile. To avoid overlaps, all profiles were vertically shifted at 0.5 intervals. The black open circles indicate the measured normalized WAXD profiles, blue broken curve is the contribution of the amorphous halo, and red curve is the summation (re-construction) of the deconvoluted peaks with the amorphous contribution.

3. Supplementary Table

Supplementary Table S1. Oligonucleotide primers. Sequences for restriction enzyme digestion necessary for ligation and sequences necessary for infusion cloning are underlined.

Primers	Sequences (5′–3′)
(For cloning)	
ser1fKpnI	TTTGGTACCAGCGGTCAGAAACCTTGTTAACC
ser1rBamHI	TTTGGATCCGTTGGCGGTCTTTGGATCGCTTG
ser3fKpnI	TTTGGTACCGCGGAGGGACTTTAGTTCTCTCT
ser3rBamHI	TTTGGATCCTTCGAAGCTTCGATTGCTCTGTCTGT
In-fusion-fSer1	GTATGGCTGACCTAGAGCGGTCAGAAACCTTGTTAACCAATAGAG
In-fusion-r1	GCCTAGTAGACCTAGCACGCGCTTGAAAGGAGTGTGTAAATGGAC
In-fusion-fSer3	GAACATTGTCAGATCTGCGGAGGGACTTTAGTTCTCTCT
In-fusion-r2	GGTTTGTCCAAACTCTCCCCTGATTCTGTGGATAACCG
In-fusion-f3xP3	GAGTTTGGACAAACCACAACTAGAATGCAGTG
In-fusion-rSV40	GCGCGCCATCGAATTCTAATTCGAGCTCGCCCGGGGATCT
(For inverse-PCR)	
INVRF	CCTCGATATACAGACCGATAAAACAC
INVRR	AACTTTTATGGCGCGCCATCGAAT
INVLF	GCGTGAGTCAAAATGACGCATGAT
INVLR	ATCAGTGACACTTACCGCATTGACA
(For qPCR)	
18Sf	GTCCGGGCCTGGTGAGATTT
18Sr	CGATCCGCCGACGTTACTACA
Ser1qf	CAAAGACCGCCAACATGCGT
Ser1qr	CACTGCTAGCTGCATTGTACTTTCG
Ser3qf	CCAGAGCCAGTCATACAACAAAG
Ser3qr	TGTCGTCGGAATTCTCACCA
